# Multi-Objective Machining Parameter Optimization Based on Strain Signal in Turning Process

**DOI:** 10.3390/mi17060658

**Published:** 2026-05-26

**Authors:** Ganggang Yin, Ze Wu

**Affiliations:** School of Mechanical Engineering, Southeast University, Nanjing 211189, China

**Keywords:** machining parameter optimization, strain signal, turning process

## Abstract

The optimization of machining parameters directly governs machining quality and efficiency. In this paper, tool holder strain signal is proposed to characterize the cutting process. It is found that the overall variation trend of the strain signal is in complete agreement with the cutting force signals, which are then used to optimize the machining parameters. Then, orthogonal cutting experiments are conducted, and the NSGA-II algorithm is used for multi-objective optimization of strain signal, surface roughness and material removal rate. The regression models derived from the orthogonal experiment results serve as the objective functions. The optimal machining parameters are obtained, with a cutting speed of 140.27 m/min, feed per rotation of 0.19 mm, and depth of cut of 1.47 mm, which offer a reference for practical production.

## 1. Introduction

The selection of machining parameters directly governs machining quality, productivity, and energy consumption [1,2,3]. During the cutting process, the cutting speed, feed per rotation, and depth of cut exert significant influences on the tool life, surface roughness, and material removal rate. Many studies have devoted a great deal of attention to their optimization to obtain the best machining effect in actual production, which is especially difficult with machine materials such as stainless steel [4,5,6]. However, these optimization objectives usually exhibit complex nonlinear interaction characteristics and conflicting trade-off relationships [7,8]. Traditional approaches, which rely on empirical trial cutting or single-objective optimization, can no longer satisfy the simultaneous demands of modern manufacturing for high precision, high efficiency, and low cost. The introduction of multi-objective optimization algorithms and the subsequent establishment of reliable machining response prediction models hold practical significance for revealing the trade-off mechanisms among multiple objectives and enabling the scientific selection of optimal machining parameters [9,10,11].

Multi-objective machining parameter optimization has been applied in various machining processes to obtain the best machining effect, such as milling, grinding and turning [12,13,14]. Yin et al. [15] applied the surface strain sensor to study the tool wear process and analyzed the relationship between the strain signal and the tool wear width. Qu et al. [16] studied thin-walled plate milling and employed NSGA-II for multi-objective optimization of the cutting force, surface roughness, and material removal rate. The regression models derived from experiments served as objective functions, and the obtained Pareto-optimal parameter combinations were validated through chatter stability analysis. Zhang et al. [17] proposed a multi-objective optimization method based on an improved Hopfield neural network (IHNN). This method integrates Pareto theory, an improved immune algorithm, and a nonmonotone activation function to optimize the machining parameters with respect to the machining energy, time, and cost. Its effectiveness was validated through comparative experiments with NSGA-II and MOPSO. Sun et al. [18] proposed a multi-objective optimization method based on NSGA-II in superalloy honeycomb core machining with ice fixation clamping to balance quality and efficiency. Models constructed from single-factor experiments and validated by ANOVA were optimized, yielding the Pareto solutions. Fan et al. [19] modeled the effects of air pressure, standoff distance, and nozzle traverse speed on material removal rate, machining width, and machining depth in abrasive air jet machining via regression analysis. The artificial bee colony algorithm was used for multi-objective optimization, and a series of Pareto-optimal solutions were successfully generated. Lin et al. [20] established optimization models for multi-pass turning under dry and wet conditions via experimental design, targeting the carbon emissions, operation time, and cost. The results obtained from the multi-objective optimization algorithm demonstrated that the use of cutting fluids can reduce carbon emissions and cost while enhancing production efficiency. Mohanta et al. [21] optimized the surface roughness and cutting force in turning by grey relational analysis, desirability function analysis, MOGA, and MOPSO. The comparative results revealed close agreement between traditional and metaheuristic optimization, and MOGA was the most efficient for this multi-criterion decision-making problem.

From the existing literature, the conventional parameter optimization method has primarily focused on cutting force, cutting temperature and surface roughness [22,23,24,25,26], while the effect of tool holder strain on machining accuracy is often neglected. In this paper, cutting process characterization based on the tool holder strain signal is proposed and verified. Through orthogonal cutting experiments, the multi-objective optimization of machining parameters in the turning process is performed using the NSGA-II algorithm, with the tool holder strain signal, surface roughness, and material removal rate serving as evaluation criteria. The optimal machining parameters are, thus, obtained, which provides a process reference for practical cutting applications.

## 2. Experimental Setup and Procedure

### 2.1. The Machining Condition Characterization Based on Surface Strain Sensor

**Figure 1:**
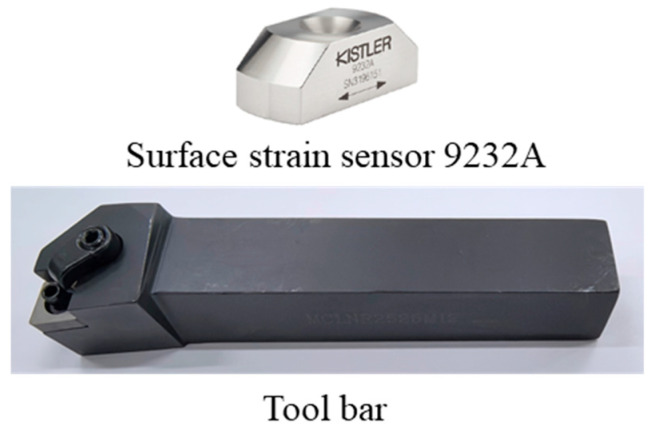
The surface strain sensor and tool holder.

The cutting force signal is a commonly used indicator for optimizing the machining parameters. However, the force sensor for measuring the force signal is expensive and inconvenient to install at the processing site. In this work, the surface strain sensor for measuring the tool holder strain signal is proposed instead of the force sensor to optimize machining parameters. The surface strain sensor employed in this work is manufactured by the Kistler Group (Type 9232A, Kistler, Winterthur, Switzerland), with a measurement range of −300 to 300 με, as depicted in Figure 1. It can be conveniently mounted onto the tool holder by using a screw, and can characterize the cutting process through the tool holder strain signal. The technical parameters of the used surface strain sensor are listed in Table 1. It presents a high natural frequency and small size close to the tool holder.

**Figure 2:**
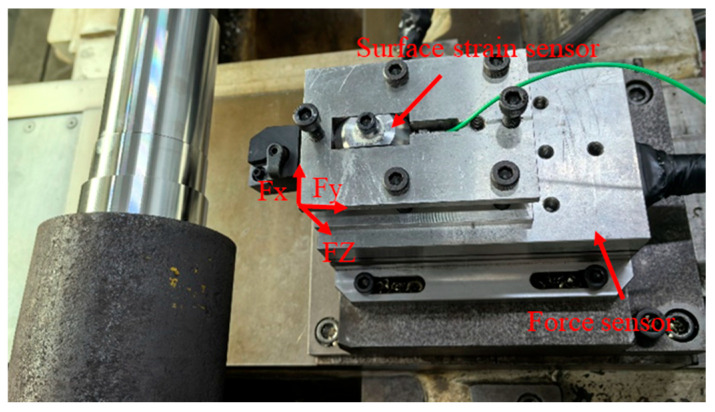
The turning experiment setup.

To verify the feasibility of the tool holder strain sensor instead of the force sensor in characterizing the cutting process, a comparison between two types of signals is first performed. For this purpose, the widely applied force sensor (9257B, Kistler, Switzerland) and strain sensor are simultaneously installed in the turning process, as shown in Figure 2. In this work, a lathe (CAK6150, Shenyang Machine Tool Co., Ltd., Shenyang, China) with a maximum spindle speed of 2200 rpm is used in the turning experiments. The workpiece material is 316 stainless steel with dimensions of ∅100 × 300 mm. The coated cemented carbide tool (CNMG120408-SM GM3225, Xiamen Golden Egret, Xiamen, China) is used to machine the 316 stainless-steel workpiece, which presents a rake angle of 15° and flank angle of 0°. The TiAlN coating and submicron grained carbide substrate of the used tool are optimized for the semi-finishing of stainless steel. In the test, the surface strain sensor is installed on the top surface of the tool holder, and the tool holder is then installed onto the force sensor via a fixture. To avoid data acquisition discrepancies, both the strain sensor and force sensor are simultaneously connected to the same charge amplifier (Type 5080A, Kistler, Switzerland) and DAQ system (Type 5697A1 Kistler, Switzerland). The sampling frequency is set to 20 kHz, which is significantly higher than the spindle rotational frequency.

**Figure 3:**
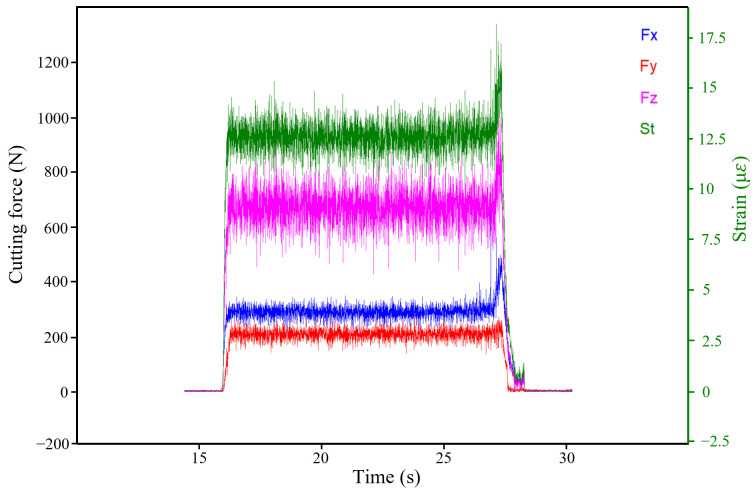
The comparison of strain signal and force signal.

The obtained strain signal and the force signals are compared under identical cutting conditions (*V_c_* = 140 m/min, *f* = 0.15 mm, *a_p_* = 2 mm), as shown in Figure 3. In the test, the strain signal *St* is measured along the axial direction on the top surface of the tool holder, while three component forces are simultaneously acquired by the force sensor, which consist of the axial force *Fx*, radial force *Fy*, and tangential force *Fz*. In comparison, the tangential force *Fz* is the largest force component in the force signal and presents a relatively large dispersion as well. When the tool contacts with the workpiece at the cut-in stage, owing to the elastic deformation of the tool holder caused by the cutting force, the strain signal first sharply rises from zero, remains steady with minor fluctuations during the stable cutting stage, and rapidly returns to zero as the tool leaves the workpiece at the cut-out stage. Due to the interference and scraping between the tool and the transition surface of the workpiece, a brief abnormal peak appears in the strain signal and force signal. Hence, the average value of the acquired signal at the stable cutting stage is adopted as the experimental result. This behavior is consistent with typical cutting processes. From the results, it is revealed that the overall variation trend of the strain signal is in complete agreement with that of the three cutting force components. This result preliminarily confirms that the strain signal is capable of characterizing the cutting process and can also be used for optimizing the machining parameters, which is directly related to the movement trajectory of the tool cutting edge. It offers a cheaper and more convenient solution compared to the force signal.

### 2.2. The Orthogonal Cutting Experiments

In the cutting process, the three primary machining parameters are the cutting speed, feed per rotation, and depth of cut. From the perspective of the machining results, the machining accuracy, surface quality, and material removal rate (MRR) are the three indicators of greatest practical concern. In rough machining, greater emphasis is placed on MRR to improve productivity, while machining accuracy and surface quality primarily affect subsequent operations. In finish machining, the focus shifts to machining accuracy and surface quality to enhance the part quality, though machining efficiency must also still be taken into account. These three cutting parameters collectively govern machining accuracy, surface quality and MRR during the cutting process. However, MRR is often in conflict with the machining accuracy and surface quality, and an excessively high material removal rate is typically accompanied by degraded machining accuracy and surface quality. In order to obtain the comprehensive optimal machining parameters, an orthogonal cutting experiment with three factors and four levels was conducted in this paper, as shown in Table 2. The factor A (cutting speed) was set at four levels ranging from 100 to 160 m/min, the factor B (feed per rotation) at four levels ranging from 0.15 to 0.3 mm, and the factor C (depth of cut) at four levels ranging from 0.5 to 2 mm. All the machining parameter ranges are selected based on the recommended combinations from the manufacturer.

The tool holder undergoes elastic deformation and generates strain and deformation under the cutting forces. Hence, the resulting strain signal can effectively represent the cutting force conditions during the machining process and is directly related to machining accuracy as well. Specifically, the strain and deformation of the tool holder cause the actual trajectory of the tool cutting edge to deviate from the ideal path, which is a primary source of machining inaccuracies. Therefore, this study proposes the strain signal as an evaluation indicator that reflects both the cutting force and machining accuracy. Various indicators are available for assessing the machined surface quality, including surface roughness, surface topography, and surface hardened layer. Among these indicators, surface roughness is the most widely used parameter in industrial practice. Accordingly, this work employs surface roughness as the evaluation indicator for the machined surface quality. In the experiments, surface roughness was measured by the handheld roughness measurement instrument (TIME3200, Beijing Time High Technology Co., Ltd., Beijing, China) along the axial direction at three different positions on the machined surface, and the average value was taken as the test result. MRR in the turning process can be calculated with the following Equation (1). The tool holder strain signal *St*, surface roughness *Ra*, and *MMR* obtained from the orthogonal cutting experiments are listed in Table 3.

(1) $$MMR=V_{c}\times f\times a_{p}$$

## 3. Results and Discussions

### 3.1. The Tool Holder Strain Signal Result Analysis

In this paper, statistical analyses, including range analysis, regression analysis and analysis of variance (ANOVA), were performed on the experimental data by the software Design-Expert 25.0. The range analysis of the strain signal in this study is presented in Table 4. Tool holder strain signal is an important indicator reflecting the force and machining accuracy during the machining process. A smaller strain signal indicates a lower cutting force and higher machining accuracy. Based on the range value of *R*, within the tested parameter range, the order of effect of the cutting parameters on the strain signal, from most significant to least significant, is the depth of cut *a_p_*, the feed per rotation *f*, and cutting speed *v*.

**Figure 4:**
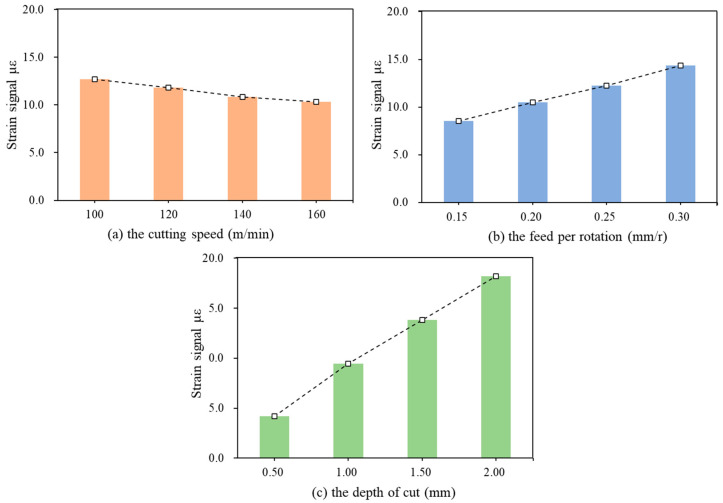
The influence of the cutting parameters on strain signal.

As illustrated in Figure 4, the tool holder strain signal tends to decrease with increasing cutting speed, but increases with higher feed per rotation and depth of cut. An increase in the cutting speed is beneficial for reducing the cutting force and thereby minimizing the dimensional errors caused by the elastic deformation of the tool holder. In contrast, increasing either the feed per rotation or the depth of cut leads to a larger cutting force, which, in turn, increases tool holder deformation and adversely affects machining accuracy. Through the range analysis, the optimal combination of parameters for minimizing tool holder strain signal is determined as the cutting speed *V_c_* = 160 m/min, the feed per rotation *f* = 0.15 mm, and depth of cut *a_p_* = 0.5 mm.

Polynomial regression is commonly used in engineering to model the functional relationship between a single dependent variable and one or more independent variables. The model parameters are fitted using the least squares method, thereby obtaining an empirical regression prediction model based on the experimental data. The multivariate quadratic polynomial regression equation is given as follows:

(2) $$\hat{y}=\beta_{0}+\sum_{i=1}^{n}\beta_{i}x_{i}+\sum_{i<j}^{n-1}\sum_{j}^{n}\beta_{ij}x_{i}x_{j}+\sum_{i}^{n}\delta_{ii}x_{i}^{2}\;\;$$

where *ŷ* is the dependent variable, *x* is the independent variable, *β* denotes the fitted coefficients, and *ε* represents the random error.

Based on the orthogonal experiment results, the fitted quadratic regression model for the strain signal *St* is as follows:

(3) $$\begin{array}{c} St=1.0178+6.2856\times a_{p}+0.41273\times f-0.042427\times V_{c}\\ \;\;\;\;\;\;\;\;\;\;\;\;\;+27.856\times f\times a_{p}-0.046091\times V_{c}\times f-0.0095091\times V_{c}\times a_{p}\\ \;\;\;\;\;\;\;\;\;\;\;\;+16.702\times a_{p}^{2}-0.85713\times f^{2}+0.0021411\times V_{c}^{2} \end{array}$$

To ensure the accuracy of the regression model, model validation was performed using ANOVA. *SS* is the sum of squares, *df* is the degree of freedom, and *MS* is the mean square of the regression model. The statistical significance of each independent variable in the model was judged by the F-value and the *p*-value. Variables with a high F-value and a *p*-value less than 0.05 were considered to have a significant influence on the dependent variable. Table 5 presents the ANOVA results for the strain signal, where *df* is the degrees of freedom, *SS* is the total sum of squares, and *MS* is the mean square. As shown in the table, the F-value of 594.88 of the fitted model is higher than $F_{0.05}\;\left(3,16\right)=8.6923$, indicating that the confidence level of the fitted model exceeds 95%. The *p*-value is less than 0.0001, indicating that the fitted model is highly significant. Comparing the F-values and the *p*-values reveals that the order of effect of the fitted terms on the strain signal is the depth of cut (factor C), feed per rotation (factor B), the interaction term BC between cutting speed and depth of cut, and the quadratic term of depth of cut (C^2^). The cutting speed (factor A) does not have a significant effect on the model. The results are consistent with the depth of cut and feed per rotation imposing significant effects on the cutting force among the machining parameters. By calculating the coefficient of determination *R*^2^ = 0.9989, this shows that 99.89% of the variability in the strain signal St is accounted for by factors A, B, C, the interaction term BC, and the quadratic term C^2^.

**Figure 5:**
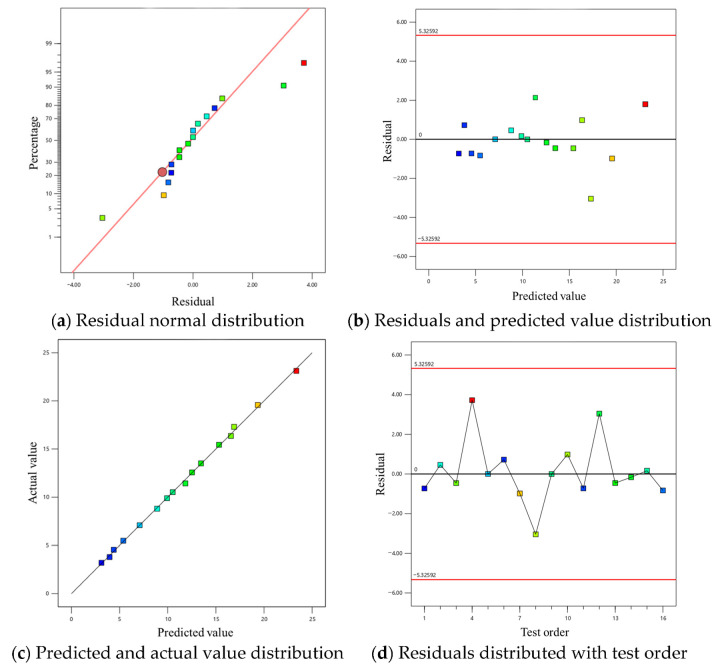
Residual analysis of the strain signal.

Residual analysis was performed on the regression model for the strain signal, as shown in Figure 5. Figure 5a presents the normal distribution of strain signal residuals. It is found that the points are distributed closely along the theoretical reference line, confirming that the residuals follow a normal distribution and that the model fits the data well. Figure 5b shows the residuals and the predicted value distribution. The results indicate that all points fall within the interval [−2, 2] and display no non-random trends or abnormal scatter. Figure 5c shows the predicted values and actual value distribution of the strain signal, where the horizontal axis represents the experimental values and the vertical axis represents the predicted values, to evaluate the prediction accuracy. It is found that most points lie tightly clustered around the *y* = *x* reference line, and no anomalous actual values are observed, indicating a satisfactory model fit. Figure 5d depicts the residuals of the strain signal against the experimental order. It is found that the residuals fluctuate randomly around zero as the test order increases, demonstrating the independence of the residuals and satisfying the model assumptions.

### 3.2. The Surface Roughness Result Analysis

**Figure 6:**
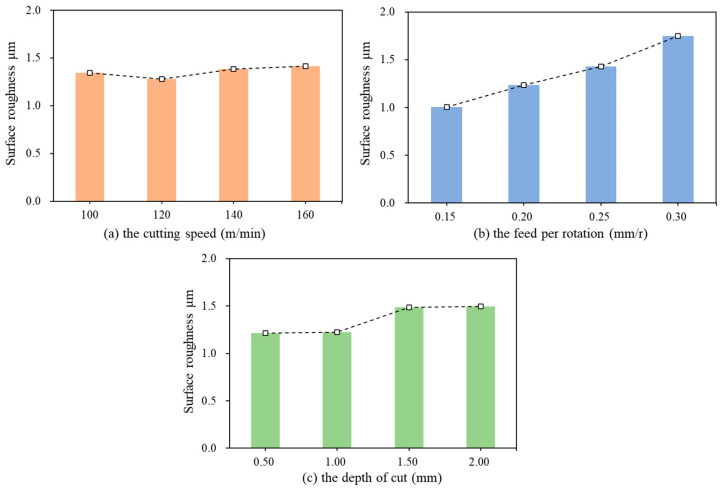
The influence of the cutting parameters on surface roughness.

Surface roughness is a widely used indicator for characterizing workpiece surface quality. According to the range analysis of surface roughness in Table 6, within the selected range of cutting parameters, the order of influence on surface roughness is the feed per rotation *f*, depth of cut *a_p_*, and cutting speed *V_c_*. As shown in Figure 6, surface roughness exhibits an increasing trend with increasing feed per rotation. The increasing feed per rotation significantly increases the height of the residual area, and also raises the cutting force and tool holder deformation. These factors intensify the rubbing between the workpiece and the tool, thereby increasing surface roughness. Surface roughness increases to a certain extent before stabilizing as the depth of cut increases. In comparison, the cutting speed has a relatively minor effect. Surface roughness exhibits fluctuation with the cutting speed, where it first decreases and then increases with increasing the cutting speed. Moderately increasing the cutting speed reduces the cutting force and suppresses the formation of built-up edge, which helps decrease surface roughness and improve surface quality. However, the excessively high cutting speed will generate more cutting heat per unit time, leading to a higher cutting temperature and accelerated tool wear, which, in turn, adversely affect surface roughness. Within the range of orthogonal experiments, the optimal machining parameter combination for minimizing the surface roughness is a cutting speed of 120 m/min, feed per rotation of 0.15 mm, and depth of cut of 0.5 mm.

Based on the orthogonal experiment results, the fitted quadratic regression model for surface roughness *Ra* is as follows:

(4) $$\begin{array}{c} Ra=-2.9515+15.539\times f+0.12079\times a_{p}+0.026596\times V_{c}\\ \;\;\;\;\;\;\;\;\;\;\;\;\;\;\;\;+2.3850\times f\times a_{p}-0.14319\times V_{c}\times f-0.0052241\times V_{c}\times a_{p}\\ \;\;\;\;\;\;\;\;\;\;\;\;\;\;\;\;+8.9418\times f^{2}+0.00063121\times V_{c}^{2}-0.0050821\times a_{p}^{2} \end{array}$$

The ANOVA results for surface roughness are presented in Table 7. From the results, it can be observed that the model has an F-value of 9.20 and a *p*-value of 0.0069, indicating that the model is statistically significant. Based on the comparison of the *p*-values, it is revealed that the feed per rotation (factor B) and the two-way interaction AB between the cutting speed and feed per rotation are the key terms in the model. The F-values indicate that the terms affect the surface roughness in the following descending order of importance: the feed per rotation (factor B) and the two-way interaction AB. By calculating the coefficient of determination *R^2^* = 0.9989, this shows that 99.89% of the variability in the surface roughness model is accounted for by factor B, the interaction term AB and the quadratic term A^2^.

**Figure 7:**
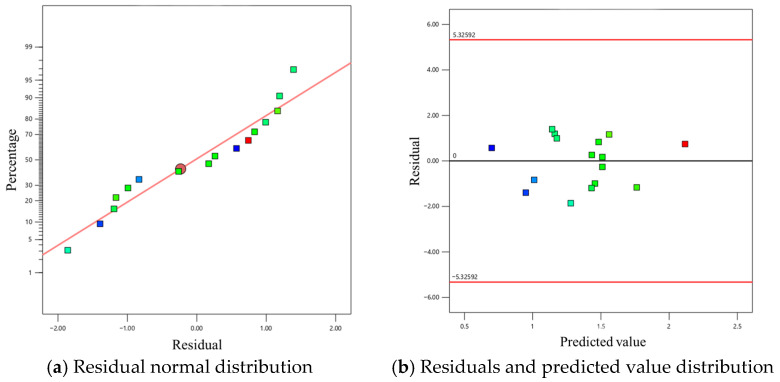

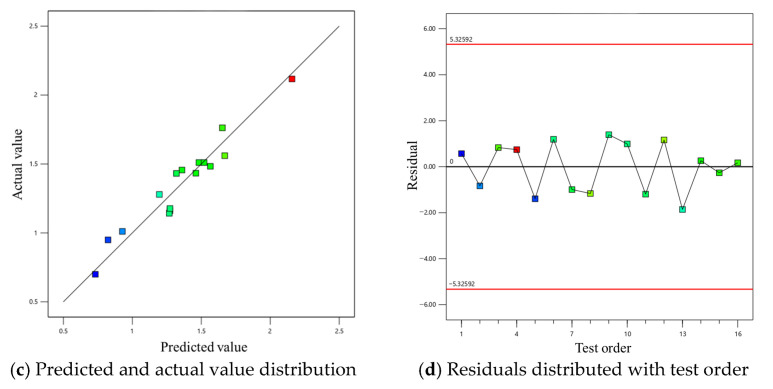
Residual analysis of the surface roughness.

The residual analysis of surface roughness is revealed in Figure 7. The prediction residuals fluctuate within a bounded range and their distribution exhibits no systematic pattern. This result confirms the validity of the surface roughness prediction model. The actual values agree closely with the fitted curve, with no outliers and relatively small relative errors, indicating that the surface roughness regression model possesses a high degree of adequacy and accuracy.

### 3.3. The Material Removal Rate Result Analysis

Traditional multi-objective optimization algorithms, such as the weighted sum method, the goal programming method and the comprehensive function method, mainly convert complex multi-objective optimization problems into single objective problems, thereby solving the original problem. However, these methods struggle to identify all the optimal solutions. Among these methods, the subjective assignment of weights to objective functions may compromise the objectivity and comprehensiveness of the obtained results. In contrast, the Non-dominated Sorting Genetic Algorithm II (NSGA-II) is an improved multi-objective evolutionary algorithm based on genetic algorithms. It employs a non-dominated sorting mechanism to approach Pareto-optimal solutions and utilizes a crowding distance metric to prevent premature convergence to local regions. NSGA-II offers high solution efficiency and good diversity of the obtained solution set. In this study, NSGA-II is adopted to obtain the Pareto-optimal solutions of machining parameters. The multi-objective optimization is formulated to minimize the strain signal and the surface roughness and maximize the material removal rate. The established regression prediction models for the strain signal and surface roughness, given by Equations (2) and (3), together with the MMR formula in Equation (1), serve as the objective functions. The constraints for the multi-objective optimization model are specified as follows:

(5) $$\left(V_{c},f,a_{p}\right)=\left(\min S_{t},\min R_{a},\max MMR\right)s.t.\left\{\begin{array}{c} 100\leq V_{c}\leq 160\\ 0.15\leq f\leq 0.3\\ 0.5\leq a_{p}\leq 2.0 \end{array}\right.$$

**Figure 8:**
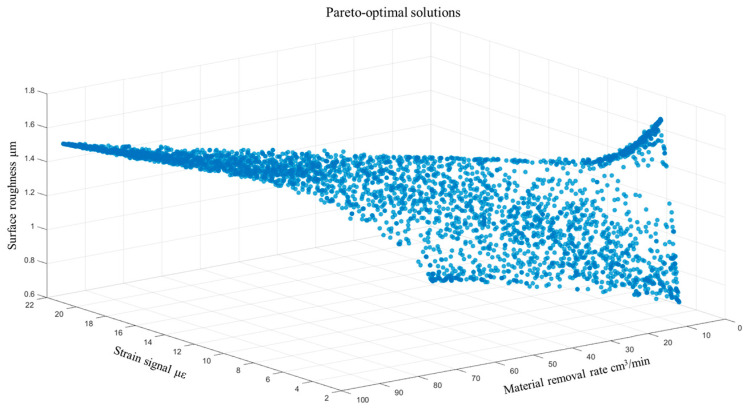
Pareto-optimal solutions surface in three-dimensional objective space.

The parameter settings of NSGA-II include the population size, crossover fraction, iteration times, and Pareto set proportion, etc. The population size represents the number of individuals simulated in each iteration and remains constant across all iteration processes. An excessively small population size leads to insufficient diversity, making it difficult to locate the optimal solution. The crossover fraction determines the position or number of crossover points, and is beneficial for increasing the population diversity. The iteration times refer to the evolutionary number that the population undergoes. Too few iteration times may cause the algorithm to fail to converge. The Pareto set proportion denotes the proportion of non-dominated solutions in the entire solution set obtained by the algorithm, with a higher proportion indicating a larger number of optimal solutions. In this study, the solver-based optimization module in the MATLAB R2025a toolbox was employed to perform multi-objective optimization. The solver algorithm was set to the Gamultiobj multi-objective optimization using the genetic algorithm. The initial population size was set to 5000 to provide an adequate number of sample points. The crossover fraction was set to 0.8 to enhance population diversity. The iteration time was set to 5000, and the Pareto set fraction was set to 0.8. The obtained Pareto-optimal solution surface in three-dimensional objective space is presented in Figure 8.

**Figure 9:**
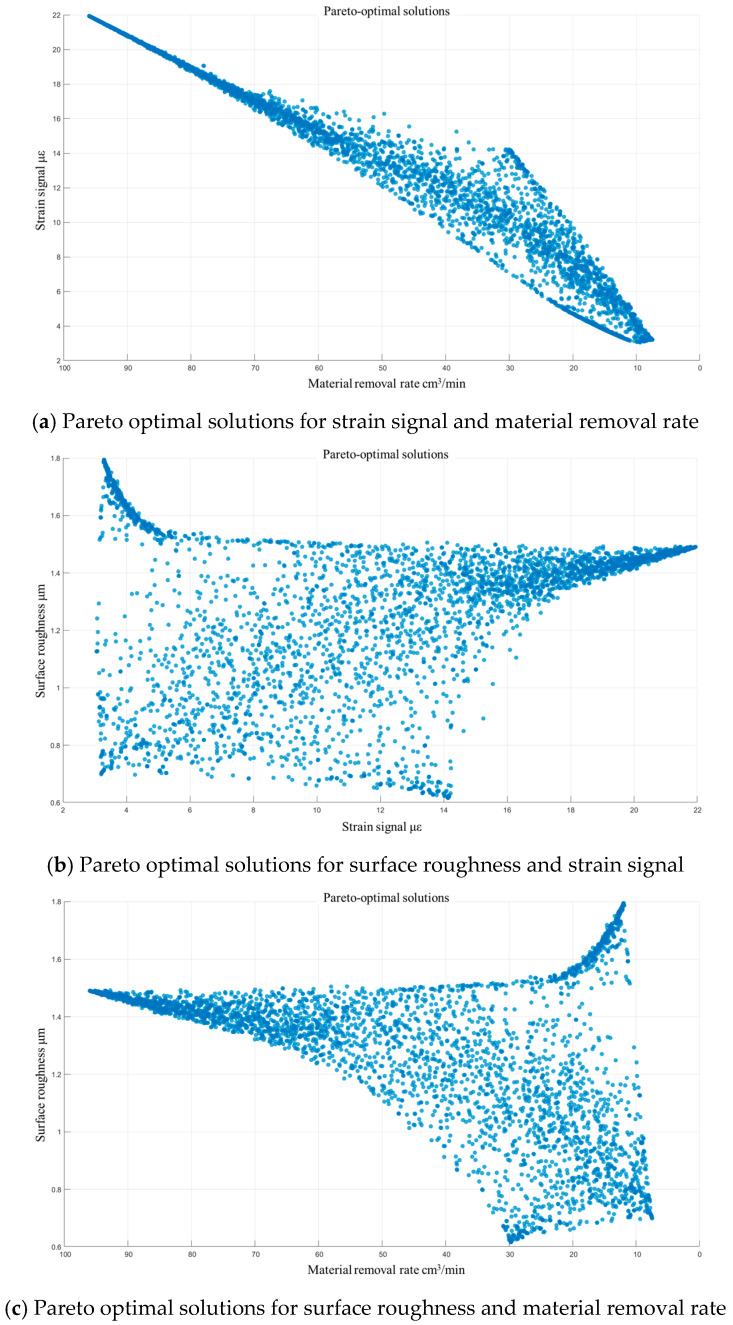
Pareto optimal solutions projected onto the coordinate planes.

In the cutting process, there exists a mutually constraining contradiction among the machining accuracy, surface roughness, and material removal rate. The obtained Pareto-optimal solutions in three-dimensional objective space were then projected onto the three coordinate planes to reveal the pairwise trade-off relationships among the optimization objectives. The Pareto-optimal solutions from the three planar projections are shown in Figure 9. As can be seen, the material removal rate exhibits a negative correlation with both strain signal and surface roughness, whereas surface roughness is positively correlated with strain signal. The Pareto-optimal solutions for strain signal and material removal rate lie on the lower boundary of the curve. Those for surface roughness and material removal rate form the lower branch of the curve. It is notable that surface roughness and strain signal can simultaneously attain their optimal values.

To balance the energy consumption and machining cost, this study considers the energy required to remove a unit volume of workpiece material. The ratio of the strain signal to material removal rate (*St/MMR*) is used as the primary focus, with a comprehensive trade-off to increase the material removal rate while reducing both the strain signal and surface roughness. A series of selected Pareto optimal solutions, along with their corresponding cutting parameters, is listed in Table 8. Based on these results, the average values of these parameters were calculated and then adopted as the optimal machining parameters: the cutting speed *V_c_* = 140.27 m/min, the feed per rotation *f* = 0.19 mm, and the depth of cut *a_p_* = 1.47 mm.

## 4. Summary and Conclusions

In this paper, turning experiments were conducted to obtain multi-objective machining parameter optimization based on the strain signal in the turning process. From the results, the following conclusions can be drawn:

1. The strain signal that was measured by the surface sensor on the tool holder is proposed to characterize the cutting process. In comparison, the overall variation trend of the strain signal was in complete agreement with the cutting force signals. This indicates that the strain signal can be used to characterize the cutting process and optimize the machining parameters, with a cheaper and more convenient approach.
2. From the range analysis of the experimental results, the order of influence of cutting parameters on the strain signal, from most significant to least significant, is as follows: the depth of cut *a_p_*, feed per rotation *f*, and cutting speed *v*. Strain signal exhibits a decreasing trend with increasing cutting speed, whereas it increases with higher feed per rotation and depth of cut. Regarding the influence on surface roughness, the order of significance is the feed per rotation *f*, depth of cut *a_p_*, and cutting speed *v.* The surface roughness first decreases and then increases with increasing cutting speed, shows an increasing trend with increasing feed per rotation, and increases up to a certain level and then stabilizes as the depth of cut increases.
3. The NSGA-II algorithm was used for multi-objective optimization on the strain signal, surface roughness and material removal rate during the cutting process. The regression models derived from the orthogonal experiment results served as the objective functions. The optimal cutting parameters were obtained: the cutting speed *V_c_* = 140.27 m/min, the feed per rotation *f* = 0.19 mm, and the depth of cut *a_p_* = 1.47 mm.

## Figures and Tables

**Table 1:** Technical parameters of surface strain sensor.

Properties	Range	Sensitivity	Natural Frequency	Linearity
Value	−300~300 με	−75 pC/με	≥12 kHz	≤±2%FSO

**Table 2:** The orthogonal cutting experiment.

	Factor	(A) *V_c_* (m/min)	(B) *f* (mm)	(C) *a_p_* (mm)
Level				
1		100	0.15	0.5
2		120	0.2	1
3		140	0.25	1.5
4		160	0.3	2

**Table 3:** The orthogonal experiment results.

No.	$V_{c}$ (m/min)	*f* (mm)	*a_p_* (mm)	*St* με	*Ra* μm	*MMR* cm^3^/min
1	100	0.15	0.5	3.12	0.73	7.5
2	100	0.2	1	8.90	0.92	20
3	100	0.25	1.5	15.33	1.57	37.5
4	100	0.3	2	23.36	2.16	60
5	120	0.15	1	7.09	0.82	18
6	120	0.2	0.5	3.96	1.27	12
7	120	0.25	2	19.36	1.36	60
8	120	0.3	1.5	16.89	1.65	54
9	140	0.15	1.5	10.52	1.27	31.5
10	140	0.2	2	16.57	1.27	56
11	140	0.25	0.5	4.40	1.32	17.5
12	140	0.3	1	11.84	1.67	42
13	160	0.15	2	13.45	1.20	48
14	160	0.2	1.5	12.53	1.46	48
15	160	0.25	1	9.93	1.48	40
16	160	0.3	0.5	5.38	1.52	24

**Table 4:** Range analysis of strain signal.

	*V_c_* (m/min)	*f* (mm)	*a_p_* (mm)
*K*	50.71	34.18	16.86
	47.30	41.96	37.76
	43.33	49.02	55.27
	41.29	57.47	72.74
*K_avg_*	12.68	8.54	4.22
	11.82	10.49	9.44
	10.83	12.26	13.82
	10.32	14.37	18.19
*R*	2.36	5.82	13.97
Optimum level	160	0.15	0.5
The order of effect on the strain signal: *a_p_* > *f* > *V_c_*			

**Table 5:** Analysis of variance of strain signal.

Source of Variance	*SS*	*df*	*MS*	*F*-Value	*p*-Value	Significant
Model	520.73	9	57.86	594.88	<0.0001	**
*A*—cutting speed	0.2254	1	0.2254	2.32	0.1788	
*B*—feed per rotation	23.78	1	23.78	244.47	<0.0001	**
*C*—depth of cut	148.15	1	148.15	1523.15	<0.0001	**
*AB*	0.0187	1	0.0187	0.1922	0.6764	
*AC*	0.0796	1	0.0796	0.8181	0.4006	
*BC*	4.27	1	4.27	43.88	0.0006	**
*A* ^2^	0.1176	1	0.1176	1.21	0.3136	
*B* ^2^	0.0279	1	0.0279	0.2867	0.6116	
*C* ^2^	0.7344	1	0.7344	7.55	0.0334	**
Residual deviation	0.5836	6	0.0973			
Total deviation	521.32	15				

** represents a significant influence on the dependent variable.

**Table 6:** Range analysis of surface roughness.

	*V_c_* (m/min)	*f* (mm)	*a_p_* (mm)
*K*	5.38	4.02	4.85
	5.11	4.94	4.91
	5.53	5.73	5.95
	5.66	7.00	5.99
*K_avg_*	1.35	1.01	1.21
	1.28	1.23	1.23
	1.38	1.43	1.49
	1.42	1.75	1.50
*R*	0.14	0.75	0.28
Optimum level	120	0.15	0.50
The order of effect on the strain signal: *f* > *a_p_* > *V_c_*			

**Table 7:** Analysis of variance of surface roughness.

Source of Variance	*SS*	*df*	*MS*	*F*-Value	*p*-Value	Significant
Model	1.7100	9	0.1898	9.20	0.0069	**
*A*—cutting speed	0.0508	1	0.0508	2.46	0.1678	
*B*—feed per rotation	0.2717	1	0.2717	13.16	0.0110	**
*C*—depth of cut	0.0021	1	0.0021	0.1009	0.7615	
*AB*	0.1804	1	0.1804	8.74	0.0254	**
*AC*	0.0240	1	0.0240	1.16	0.3222	
*BC*	0.0313	1	0.0313	1.52	0.2644	
*A* ^2^	0.0102	1	0.0102	0.4934	0.5088	
*B* ^2^	0.0080	1	0.0080	0.3873	0.5566	**
*C* ^2^	0.0000	1	0.0000	0.0013	0.9729	
Residual deviation	0.1239	6	0.0206			
Total deviation	1.8300	15				

** represents a significant influence on the dependent variable.

**Table 8:** The selected Pareto optimal solutions and their cutting parameters.

No.	*V_c_* (m/min)	*f* (mm)	*a_p_* (mm)	*St* με	*Ra* μm	*MMR* cm^3^/min
1	140.740	0.188	1.495	12.023	1.228	39.458
2	140.389	0.178	1.614	12.508	1.179	40.423
3	140.243	0.193	1.323	10.878	1.263	35.725
4	140.089	0.174	1.573	12.034	1.175	38.400
5	139.921	0.210	1.324	11.556	1.300	38.962

## Data Availability

Data are contained within the article.
